# *TLR4* T399I Polymorphism and Endometriosis in a Cohort of Italian Women

**DOI:** 10.3390/diagnostics10050255

**Published:** 2020-04-27

**Authors:** Enrica Marchionni, Maria Grazia Porpora, Francesca Megiorni, Ilaria Piacenti, Agnese Giovannetti, Cinzia Marchese, Pierluigi Benedetti Panici, Antonio Pizzuti

**Affiliations:** 1 Department of Experimental Medicine, Sapienza University of Rome, Policlinico Umberto I Hospital, 00161 Rome, Italy; 2 Department of Maternal and Child Health and Urology, Sapienza University of Rome, Policlinico Umberto I Hospital, 00161 Rome, Italy; 3 Laboratory of Clinical Genomics, Fondazione IRCCS Casa Sollievo della Sofferenza, 71013 San Giovanni Rotondo, Italy

**Keywords:** endometriosis, TLR4, T399I polymorphism

## Abstract

Background: Endometriosis is a widespread multifactorial disease in which environmental, genetic, and epigenetic factors contribute to the phenotype. Single Nucleotide Polymorphisms (SNPs) in genes implicated in pivotal molecular mechanisms have been investigated as susceptible risk factors in distinct populations. Among these, Toll-like receptor 4 (TLR4) represents a good candidate due to its role in the immune/inflammatory response and endometriosis pathogenesis. Methods: The *TRL4* gene T399I SNP (C/T transition, rs4986791) was investigated in 236 Italian endometriosis patients and 150 controls by using the PCR-RFLP method. One-tailed Fisher’s exact test was used to compare differences between categorical variables. T399I genotype distribution was evaluated for Hardy–Weinberg equilibrium in both groups using the Chi-squared test for given probabilities. Results: Fisher’s exact test comparing C and T allele frequencies showed a difference in the frequency of T alleles between patients and controls (OR = 1.96, 95% confidence interval 0.91–4.23; *p*-value = 0.0552). Genotype frequencies did not show any significant difference between patients and controls. The homozygous TT genotype was observed in 2% of endometriosis women and not in controls. Conclusions: Our results show that the *TLR4* rs4986791 T variant may be considered a genetic risk factor for endometriosis in Italian women. More extensive studies in other populations are needed to confirm this result.

## 1. Introduction

Endometriosis is a gynecological condition characterized by the presence of ectopic endometrial tissue (endometrial glands and stroma) predominantly in the pelvic peritoneum, ovarian and rectovaginal septum and, more rarely, in the diaphragmatic, pleural, and pericardial spaces [1]. The disease is a common health problem affecting up to 10% of women in reproductive age [2]. The phenotypic manifestations vary from a mild disease with peritoneal involvement to a severe form with both affected ovaries, infiltrating lesions and extensive adhesions [3]. Symptoms are often accompanied by chronic pelvic pain, dysmenorrhea, dyspareunia and reduced fertility [4]. Intensity of pain symptoms varies significantly among patients, not always correlated with the severity of endometriosis. In particular, in women with chronic pelvic pain, psychological consequences may arise, also influencing the perception of pain [5,6]. Endometriosis is a multifactorial disorder in which environmental, pro-inflammatory, genetic, and epigenetic factors may trigger the specific clinical signs [7].

Twin studies estimated endometriosis heritability at approximately 50% [8], however, its pathogenesis and pathophysiology have not been fully elucidated so far, although many studies have been conducted to investigate the innate and acquired risk factors. 

Many endometriosis susceptibility genes have been detected by Genome Wide Association Studies (GWAS) [9]. GWAS, however, cannot identify genetic specific associations in different populations [10], since there are differences in terms of genetic susceptibility and disease manifestations, as demonstrated by genetic association studies in selective populations [11]. 

The role of Toll-like receptors (TLRs), which regulate the activation of immune response and inflammation pathways, has been largely investigated in endometriosis [12]. Altered Toll-like receptor 4 (TLR4) expression or function in endometrium was demonstrated in many in vitro and in vivo studies [13,14,15,16]. TLR4 is expressed in macrophages, endometrial and endometriotic epithelial cells as well as in stromal cells [17], and it is implicated in the response to exogenous (i.e., Lipopolysaccharide, LPS) [18] or endogenous ligands (i.e., low-density lipoprotein and heat shock proteins) [19]. Recent studies pointed out the involvement of the LPS/TLR4 cascade in the growth regulation in endometriosis tissues [17], since LPS/TLR4 dysfunction may impair the correct mechanism of immune response and favor the onset and progression of the disease. Most studies in different infectious, inflammatory, and autoimmune diseases have focused on two Single Nucleotide Polymorphisms (SNPs) (D299G, rs4986790 and T399I, rs4986791) within the *TLR4* gene (OMIM * 603030), which have been demonstrated to be (or not) susceptible genetic factors in different populations [20]. The first SNP is an A/G transition causing an Asp/Gly polymorphism at amino acid 299 (D299G; rs4986790) and the second one is a C/T transition causing a Thr/Ile polymorphism at amino acid 399 (T399I; rs4986791). The *TLR4* D299G SNP has been previously investigated in endometriosis, in Indian [21] and Brazilian [22] cohorts of women. The *TRL4* T399I polymorphism, however, has never been investigated in endometriosis patients. The aim of the present study was to clarify the role of the *TRL4* T399I polymorphism in this complex genetic scenario, by performing a case-control study comprising an Italian cohort of 236 patients affected by endometriosis and 150 healthy controls.

## 2. Materials and Methods 

### 2.1. Patients and Controls

A total of 236 Italian unrelated patients were recruited from the Department of Maternal and Child Health and Urology of Policlinico Umberto I Hospital, Sapienza University of Rome, and were included in the study. All were affected by endometriosis with diagnosis confirmed by laparoscopy and histologic analysis. Stages of disease were classified according to the revised American Society for Reproductive Medicine (rASRM) classification [23]. In the study group, dyspareunia, dysmenorrhea and pelvic pain were investigated using VAS and assigning a score based on the intensity perceived by the patient (0: no pain; 1–4: mild; 5–7: moderate; 8–10: severe). As controls, 150 healthy women from the same ethnic origin were enrolled in the study. Pelvic examination and transvaginal ultrasound were performed and resulted normal. Laparoscopy was not performed systematically but, when available, resulted normal in these women. 

All subjects gave their informed consent for inclusion before they participated in the study. The study was conducted in accordance with the Declaration of Helsinki, and the protocol was approved (reference code 3545/2015) by the Ethics Committee of The Institutional Review Board of Policlinico Umberto I Hospital.

### 2.2. DNA Extraction and T399I Genotyping

Genomic DNA was extracted from 5 mL of peripheral whole-venous blood using a salting out procedure, as previously described [24], and quantified by NanoDrop 2000 (Thermo Fisher Scientific, Inc., Waltham, MA, USA). T399I polymorphism (+1196C/T transition; rs4986791) was typed by the polymerase chain reaction-restriction fragment length polymorphism (PCR-RFLP) method. Briefly, the DNA region comprehending the polymorphism was amplified by PCR by using the TLR4 T399I-forward: 5′-GGTTGCTGTTCTCAAAGTGATTTTGGGAGAA-3′ and TLR4 T399I-reverse: 5′-ACCTGAAGACTGGAGAGTGAGTTAAATGCT-3′ primers. Amplifications were carried out in a final volume of 30 μL including 200 ng of DNA template, primers at 500 nM and 1 U of Taq polymerase (Invitrogen, Carlsbad, CA, USA). PCR conditions were as follows: denaturation for 4 min at 95 °C, 35 cycles of 45 sec at 95 °C, 45 sec at 67 °C and 90 sec at 72 °C, followed by 5 min at 72 °C. The specific amplificated band of 406 bp was digested by a HinfI enzyme (New England Biolabs, Ipswich, MA, USA), following the manufacturer’s instructions. PCR-RFLP products were separated upon digestion in 3% NuSieve GTG agarose gels (Lonza Rockland, Rockland, ME, USA) with GelRed Nucleic Acid Gel Stain (Biotium, Fremont, CA, USA) and visualized under Ultraviolet (UV) light by using a ChemiDoc XRS analyzer (Bio-Rad, Hercules, CA, USA). 

### 2.3. Statistical Analysis

All statistical analyses were performed with R: A Language and Environment for Statistical Computing (R Core Team, R Foundation for Statistical Computing, Vienna, Austria, 2019) and a *p*-value < 0.05 was considered statistically significant. The Fisher’s exact test was used to compare differences between categorical variables. Odds ratio (OR) and 95% confidence interval were calculated. T399I genotype distribution was evaluated for Hardy–Weinberg equilibrium in the control group and in the patient group by Chi-squared (χ^2^) test for given probabilities. 

## 3. Results

To investigate whether the *TRL4* T399I polymorphism (rs4986791) is associated with endometriosis in the Italian population, we performed a case-control study, comparing 236 unrelated patients with 150 healthy women. The mean age in the endometriosis group was 41 years ± 9 SD. According to the Visual Analogue Scale (VAS), severe or moderate dysmenorrhea was respectively present in 50.3% and 25.1% of women with endometriosis, moderate or severe dyspareunia in 16.2% and 22.0% and moderate or severe chronic pelvic pain in 15.7% and 14.7%. Only 17.9% of these patients were affected by infertility. According to the rASRM classification [23], 2.1% of women had stage I endometriosis, 7.2% had stage II, 54.7% had stage III and 36.0% had stage IV. Healthy controls (*n* = 150) had a mean age of 35.2 years ± 11 SD and did not present a history of endometriosis nor clinical and ultrasonographic signs of this disease. A polymerase chain reaction (PCR) amplicon of 406 bp was obtained, which resulted indigested upon incubation with a HinfI restriction enzyme in the presence of C nucleotide, whilst was cut in two fragments of 378 and 28 bp when the allele T was present (data not shown).

Frequency of the C allele resulted 94 % among patients and 97 % in controls and the frequency of the T allele resulted 6% among patients and 3% in controls. The one tailed *p*-value of the Fisher’s exact test comparing the frequencies of the two alleles was *p* = 0.0552, showing a slight statistical evidence of difference between endometriosis patients and controls. The OR resulted 1.96 (95% confidence interval 0.91–4.23). The frequency of the three different genotypes did not show any significant differences between patients and controls, however, TT genotype was observed only in women with endometriosis (2%). Table 1 summarizes the allele and genotype frequencies of the *TRL4* T399I polymorphism in affected and healthy women. 

In an inheritance model, in which T is the dominant allele and the risk factor for endometriosis, the prevalence of CT heterozygotes and TT homozygotes, collectively considered, resulted higher in affected women than controls (10% vs. 6.0%) but this difference was not statistically significant (Table 1). 

The four TT homozygotes, identified in endometriosis patients only, presented advanced stages of disease according to the rASRM classification [23], two patients in stage III and two in stage IV. Other details on clinical phenotype including VAS, age, Body Mass Index (BMI), other associated diseases and infertility are reported in Table 2. Mean age in homozygous patients (TT genotype) was 43.5 years ± 9 SD.

The genotype distribution resulted in Hardy–Weinberg equilibrium (HWE) in controls but not in patients. 

In the control group, the expected frequency of the three genotypes was 94% CC, 6% CT, 0% TT and in the observed group was 94% CC, 6% CT, 0% TT (χ^2^ = 0.14348, d.f. = 2, *p*-value = 0.9308). The *p*-value is higher than 0.05, suggesting no deviation of HWE in the control group. 

In the endometriosis group, the expected frequency of the three genotypes was 89% CC, 11% CT, 0% TT, whereas in the observed group was 90% CC, 8% CT, 2% TT (χ^2^ = 15.179, d.f. = 2, *p*-value = 0.0005057). 

Therefore, in the endometriosis group a significant deviation from the HWE (*p*-value = 0.0005057) was observed, suggesting that differences are not attributed to chance. 

Altogether, our results indicate that the presence of the *TLR4* rs4986791 T variant may be considered a genetic risk factor for endometriosis.

## 4. Discussion

Endometriosis is an insidious disorder, often labeled as the “missing disease” [25], with an important diagnostic delay and still lack of resolutive treatments, significantly impacting women’s daily lives. It represents a chronic disease, inducing pain and psychological involvement, with negative consequences affecting fertility, sexuality, ability to work, and social and personal relationships [4]. Many women feel that endometriosis controls and limits their lives. Moreover, the need of long-term treatments and the absence of a definitive cure can be contributing factors. The impact of endometriosis is worsened by a lack of understanding of the disease’s causes and an increased awareness is needed in society and in research to elucidate its etiopathogenesis.

As for other multifactorial diseases, symptoms and causes of endometriosis are related to an aberrant interplay among genetic, epigenetic, and environmental modifications. The study of genetic backgrounds in different ethnic populations is crucial to better understand these complex interactions. In the last decade, many gene polymorphisms implicated in different molecular pathways have emerged as good candidates to be investigated. Considering the increasing evidence of an inappropriate immunological response in the pathogenesis of several complex disorders, many studies have been conducted to identify the genetic determinants that can modify the immune system. Among these, SNPs in the coding and promoter regions of the human *TLR4* gene have been demonstrated to induce a dysregulated TLR4 signaling pathway, this directly correlated to various infectious, autoimmune, allergic, inflammatory diseases, and lastly, endometriosis [20].

Indeed, TLR4 is essential for the innate immune response and it is composed of three domains: an extracellular domain mediating LPS recognition and receptor dimerization, a transmembrane domain and Toll/Interleukin-1 receptor (TIR) domain, which is essential for downstream signal transduction [26]. For the LPS recognition, the extracellular domain of TLR4 is associated with myeloid differentiating protein 2 (MD2), forming a complex and conferring LPS responsiveness to TLR4 [27]. Given its importance in pathogen recognition, TRL4 is highly polymorphic even if its structure is conserved [28]. The extracellular domain shows many sequence variations, such as the D299G and T399I changes, caused by two nonsynonymous and commonly co-segregating SNPs in Caucasian populations [29]. D299G change causes LPS hypo-responsiveness and markedly reduced expression of the protein on the surface of epithelial cells, and T399I change shows a milder LPS-hyporesponsive phenotype [30]. The combined effect of the two SNPs leads to the most severe LPS hypo-responsiveness compared to the responses caused by the single alleles [30]. Consequently, a debated question is how D299G and T399I changes may affect the LPS responsiveness. Preliminary studies have been shown to affect appropriate interaction with agonist or coreceptor [31] or suppressing activation of transcription factors [32]. Crystallography showed that the D299G change may locally disrupt the TLR4 structure, while T399I does not affect the wild type and mutant TLR4 structure [33]. More recent computational approaches of molecular dynamics show that both polymorphisms may abrogate the stability of the hexamer complex, leading to a compromised TLR4 signaling [34]. The role of the two *TLR4* SNPs has been largely investigated in many diseases, with genetic association to some infection-related susceptibility, such as Gram-negative caused diseases and septic shock [35,36] or to inflammatory diseases susceptibility, such as Crohn’s [37]. Negative studies have been also reported, i.e., in Systemic Lupus Erythematous [38].

In endometriosis patients, a significant increase in TLR4 mRNA expression in ectopic endometriotic lesions compared with corresponding eutopic tissue was reported [13]. Hayashi et al. [15] studied TLR4 expression in endometriosis patients, confirming an increased expression of the TLR4 mRNA and protein in ectopic endometrium compared to eutopic endometrium. A higher expression of TLR4 mRNA was also observed in the menstrual phase in the eutopic endometrium of healthy women, probably as a defense mechanism of the uterus. Both menstrual blood and shed endometrium could be a favorable environment for bacterial infection [15]. 

The role of the *TRL4* D299G polymorphism in determining susceptibility to endometriosis has been previously investigated in two different cohorts of women affected by endometriosis. The first study demonstrated an association between the GG genotype and the G allele and the risk of endometriosis in an Indian cohort [21]. Authors hypothesized that in women carrying the *TLR4* D299G polymorphism, particularly during the period of menstruation, the retrogradely shed endometrial cells could be implanted on the peritoneum (in the pouch of Douglas) and favorite the onset of endometriosis [21].

The second study, conducted in a Brazilian cohort, did not show significant differences in genotypic or allelic frequencies between patients and controls groups, with no individuals carrying the GG genotype [22]. This discordant finding could be attributed to the different constitution of the two populations, as the worldwide frequencies of *TLR4* polymorphisms show distinct patterns of distribution considering different populations and ethnic groups [39].

In the present study, we used a case-control approach to assess if *TLR4* T399I polymorphism is a susceptibility factor to endometriosis in Italian women. Our data suggest an association of the T allele with endometriosis disease, showing a 1.96-fold increased OR when evaluating patients and controls. Interestingly, homozygous TT genotype was only observed in women with endometriosis, but not in healthy females, even if the comparison did not result significant. However, considering the frequency of endometriosis and the Minor Allele Frequency (MAF) of the *TLR4* T399I SNP, the lack of significant association between genotype and endometriosis risk may be due to the relatively low number of analyzed women, this representing the main limit of the present research. More extensive studies, also involving other genetic variations in the *TLR4* gene as well as replication in larger number of patients and in different populations, will be needed to confirm our observations. Interrogation of the Genome Aggregation Database (gnomAD, v.2.1.1), the largest population database to date, in 140,863 available individuals, T399I shows an overall MAF of 6%, ranging from 1% in Africans to 6% in Europeans (non-Finnish) and 10% in Finnish. In gnomAD, TT homozygotes are very rare (0.4%). On the contrary, in our cohort of patients, homozygotes have a frequency of almost 2% vs. 0% in the control group. 

The four TT homozygous patients did not show a specific association neither with higher VAS scores for dyspareunia, dysmenorrhea and pelvic pain, nor with infertility (Table 2). Nonetheless, all the TT patients presented advanced stages of endometriosis (III and IV, Table 2), this suggesting that the T399I polymorphism might be involved in the progression and severity of the disease but future studies and functional analyses are needed to demonstrate our hypothesis.

Table 3 summarizes some details on the genetic relationship between the *TLR4* gene and endometriosis in Indian [21], Brazilian [22] and Italian populations (present research).

In previous studies of Italian cohorts of endometriosis patients, environmental factors [40], as well as different SNPs in other genes were investigated [41,42,43,44]. More recently, in a Mediterranean cohort of patients (Sardinian population), no significant association was observed for three SNPs present in *WNT4*, *VEZT* and *FSHB* genes (rs7521902, rs10859871 and rs11031006, respectively), which have been previously identified as risk factors for endometriosis in other populations [45]. Both negative and positive results are important to shed light on risk factors of this complex disease. 

According to our observation, the *TLR4* polymorphisms and the related inflammation pathways seem to be good candidates to further investigations. Many studies have linked genetic variations in *TLR4* with susceptibility to different infectious and inflammatory diseases, with complex interactions depending on environmental factors, ethnic backgrounds, and multigenic effects [20], probably involving different gene networks.

The identification of genetic risk factors in specific populations may better elucidate their effects and their possible roles in disease prognosis and prevention [46]. 

To date, in endometriosis patients, diagnosis delays and limited effectiveness of treatments are well known issues, causing negative impacts on women’s quality of life. In multifactorial diseases such as endometriosis, the genetic contribution to the phenotype depends on the combination of alleles, and multiple genetic interactions in different pathways contribute to disease susceptibility and possibly correlate with specific phenotypes in terms of disease severity, progression and associated symptoms. Identifying genetic risk factors could improve the management and prevention of physical and psychological complications of a specific disorder. 

In conclusion, our data indicate the association of the *TLR4* rs4986791 T allele with endometriosis in an Italian cohort of women, this supporting the role of genetic variations in the modulation of the TLR4-mediated immune response in endometriosis disease. 

## Figures and Tables

**Table 1:** Allele and genotype frequencies of the *TLR4* T399I polymorphism in patients with endometriosis and controls. The genetic model was considered dominant in the hypothesis that the T variant is a risk factor for the disease.

*TLR4*	Patients (236)		Controls (150)	
	n %		n %	
**Allele**				
**C**	445	(94%)	291	(97%)
**T**	27	(6%)	9	(3%)
**Genotype**				
**CC**	213	(90%)	141	(94%)
**CT**	19	(8%)	9	(6%)
**TT**	4	(2%)	0	(-)
		
**Dominant model**				
**CT+TT**	23	(10%)	9	(6%)
**CC**	213	(90%)	141	(94%)

**Table 2:** Clinical characteristics of patients with TT genotype at TLR4 polymorphism.

Patient ID	Age	BMI	Dyspareunia *	Dysmenorrhea *	Pelvic Pain *	Infertility	Associated Diseases	Surgery	Stage **
1	35	21.1	mild	moderate	no	no	no	monolateral endometriosic cyst	IV
2	54	18.4	no	moderate	no	no	Multiple Sclerosis	monolateral endometriosic cyst	IV
3	37	22.2	severe	severe	severe	no	Irritable Bowel Syndrome	monolateral endometrioma and adenomyosis	III
4	48	20	mild	no	no	no	Gestational Diabetes	monolateral endometrioma and peritoneal endometriosis	III

* Dyspareunia, dysmenorrhea and pelvic pain were investigated using VAS and assigning a score based on the intensity perceived by the patient (0: no pain; 1–4: mild; 5–7: moderate; 8–10: severe). ** Stages of disease were classified according to the rASRM classification [23].

**Table 3:** *TLR4* SNPs in endometriosis patients and controls in different populations.

*TLR4* SNPs [Ref]	Endometriosis Group	Control Group	*p*-Value (χ^2^)	Odds Ratio (Confidence Interval)
rs4986790 A/G [21] Indian population	*N* = 200	*N* = 200		
Allele frequency	A = 89% G = 11%	A = 97% G = 3%	*p* < 0.05	OR = 4.48 (95% CI 2.28–8.80)
rs4986790 A/G [22] Brazilian population	*n*= 100	*n*= 100		
Allele frequency	A = 96% G = 4%	A = 95% G = 5%	ns	ns
rs4986791 C/T present study, Italian population	*n* = 236	*n* = 150		
Allele frequency	C = 94% T = 6%	C = 97% T = 3%	*p* = 0.0552	OR = 1.96 (95% CI 0.91–4.23)

## Abbreviations

SNP	Single Nucleotide Polymorphism
TLR4	Toll-like receptor 4
C/T	Cytosine to Thymine
PCR-RFLP	Polymerase Chain Reaction-Restriction Fragment Length Polymorphism
Thr/Ile	Threonine to Isoleucine
T399I	Threonine 399 to Isoleucine
OR	Odds Ratio
GWAS	Genome Wide Association Study
TLRs	Toll-like receptors
LPS	Lipopolysaccharide
LPS/TLR4	Lipopolysaccharide/Toll-like receptor 4
A/G	Adenine to Guanine
Asp/Gly	Aspartate to Glycine
D299G	Aspartate 299 to Glycine
SD	Standard Deviation
bp	base pair
HWE	Hardy–Weinberg equilibrium
ASRM	American Society for Reproductive Medicine
BMI	Body Mass Index
VAS	Visual Analogue Scale
TIR	Toll/Interleukin-1 receptor
MD2	Myeloid Differentiating protein 2
MAF	Minor Allele Frequency
gnomAD	Genome Aggregation Database
WNT4	Wingless-Type MMTV Integration Site Family Member 4 gene
VEZT	Vezatin gene
FSHB	Follicle-Stimulating Hormone Beta Polypeptide gene
UV	Ultraviolet
